# Supplementary material for: Nucleosome dynamics of human iPSC during neural differentiation
Source: EMBO Rep. 2019 Apr 29;20(6):e46960. doi: 10.15252/embr.201846960 (PMC6549019; doi:10.15252/embr.201846960)
Supplement: Supplementary file 1 — Expanded View Figures PDF [file EMBR-20-e46960-s001.pdf]

Expanded View Figures

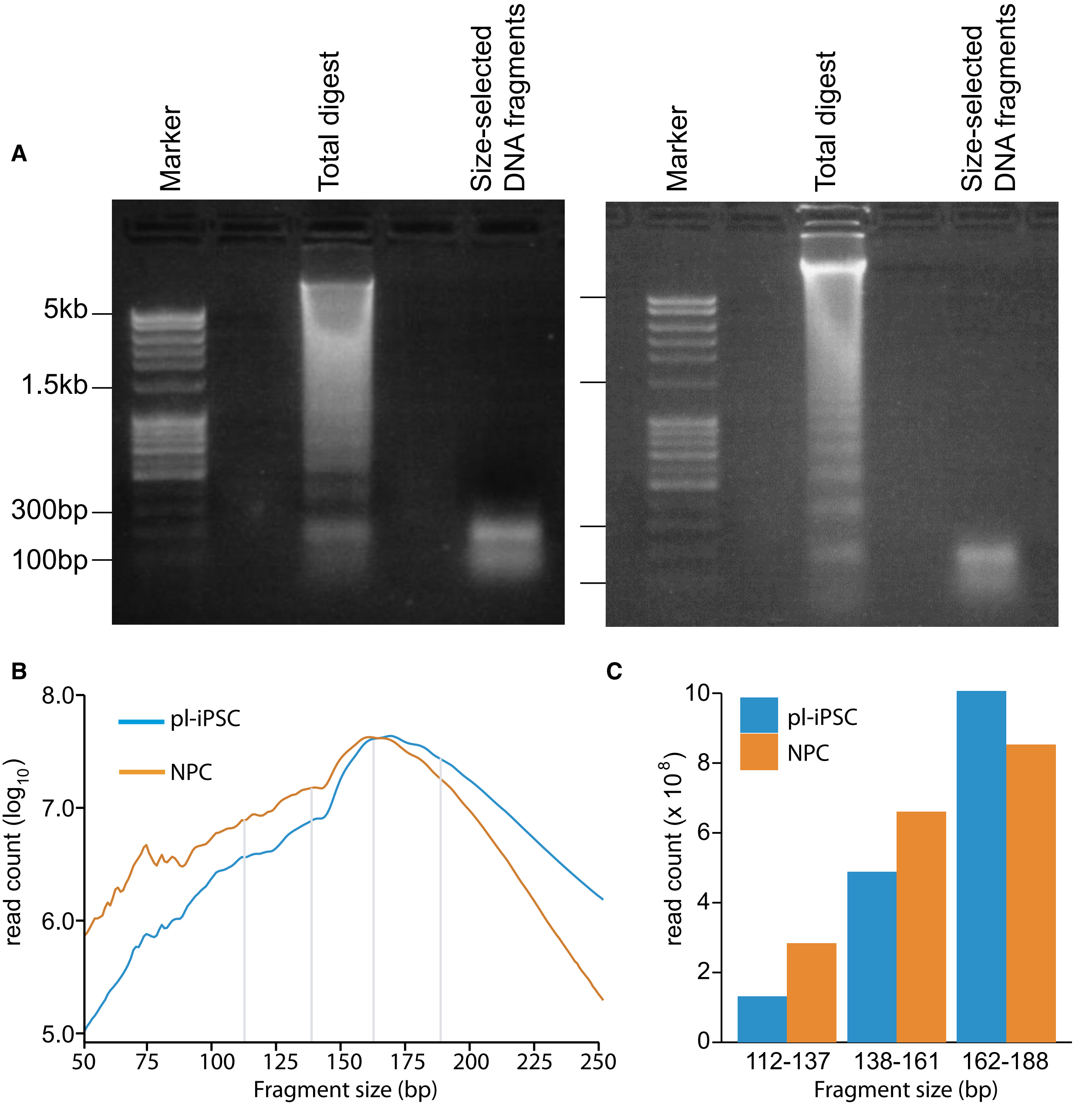

Figure EV1. Size distribution of MNase-digested fragments.

A Agarose gel (1%) images showing matched MNase digests from bulk human chromatin from pl-iPSC (left panel) and NPC (right panel), and the subsequent size-selected DNA fragment range used for paired-end sequencing. Gels were scanned and the mono-nucleosome peak quantified as a fraction of the total chromatin DNA. MNase digestion released near equivalent amounts of mono-nucleosome DNA, pl-iPSC (3% of total DNA) and NPC (2.4% of total DNA). This indicates approximately equal numbers of nucleosomes in both cell types, but we subsequently show that only a small fraction of these are positioned within the chromatin.

B The size distribution of fragments calculated from the pl-iPSC and NPC paired-end sequencing (from a total of 3.4 and 3.0 billion paired-end reads for pl-iPSC and NPC, respectively). NPC samples had a slightly smaller fragment size distribution, with no evidence for loss of 139–161 fragments (corresponding to core nucleosomes) from pl-iPSC samples.

C Histogram to compare the key size ranges of 112–137 bp (sub-nucleosome), 138–161 bp (core nucleosome) and 162–188 bp (large nucleosome footprint) for pl-iPSC and NPC samples (delineated by grey lines in B).

© 2019 The Authors

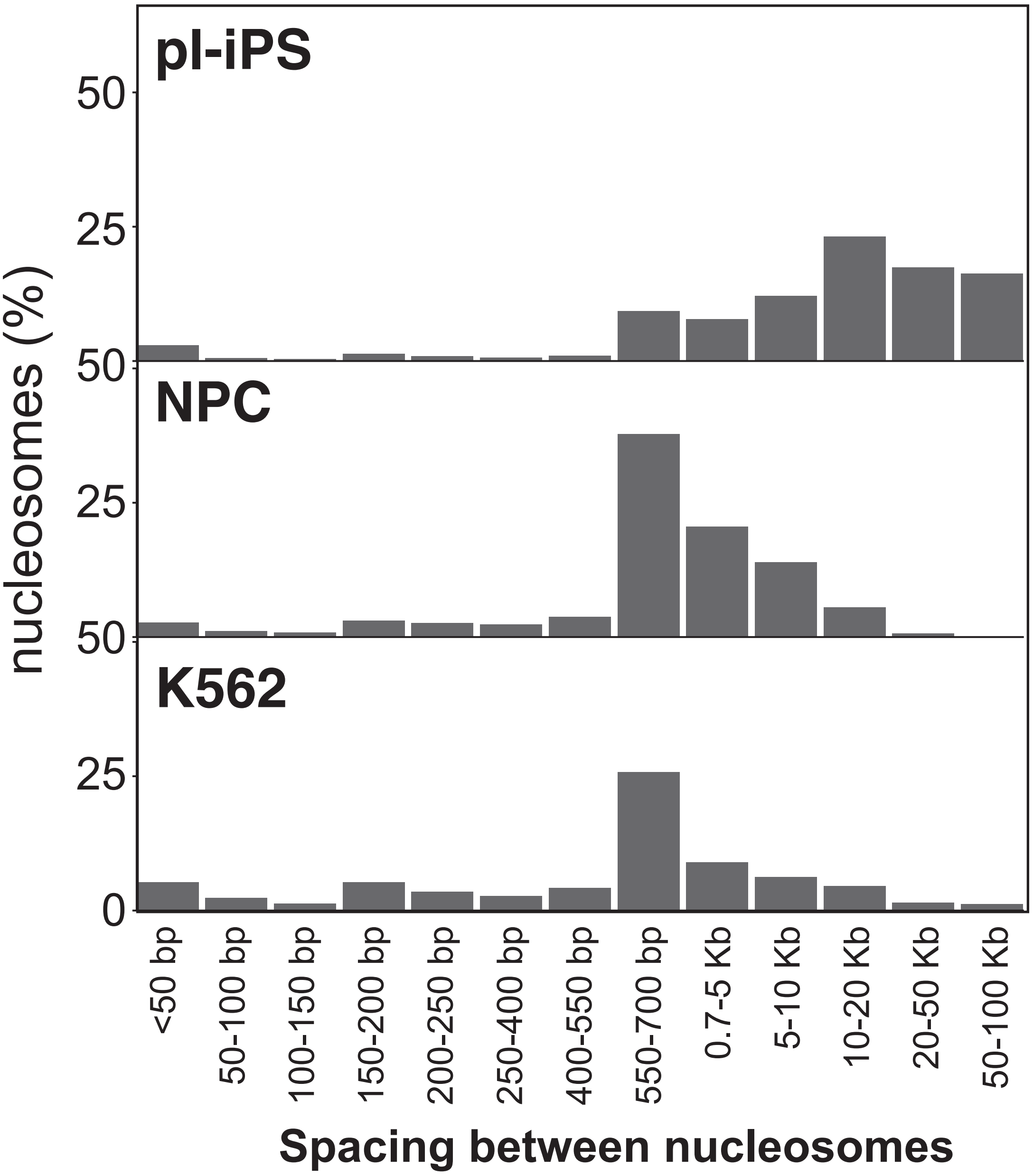

Figure EV2. Distribution of inter-nucleosome spacing.
The distance between nucleosomes (also known as linker length) was plotted between each adjacent nucleosome for pl-iPSC, NPC and K562 cells. In all three cases, the distribution of inter-nucleosome spacing distances is bimodal, with a small peak at < 50 bp, representing only a small proportion on nucleosomes, with a second peak in the range of 10–20 kb for pl-IPSC or 550–700 bp range for the differentiated NPC and K562 cells, which possess eightfold more positioned nucleosomes.

 © 2019 The Authors

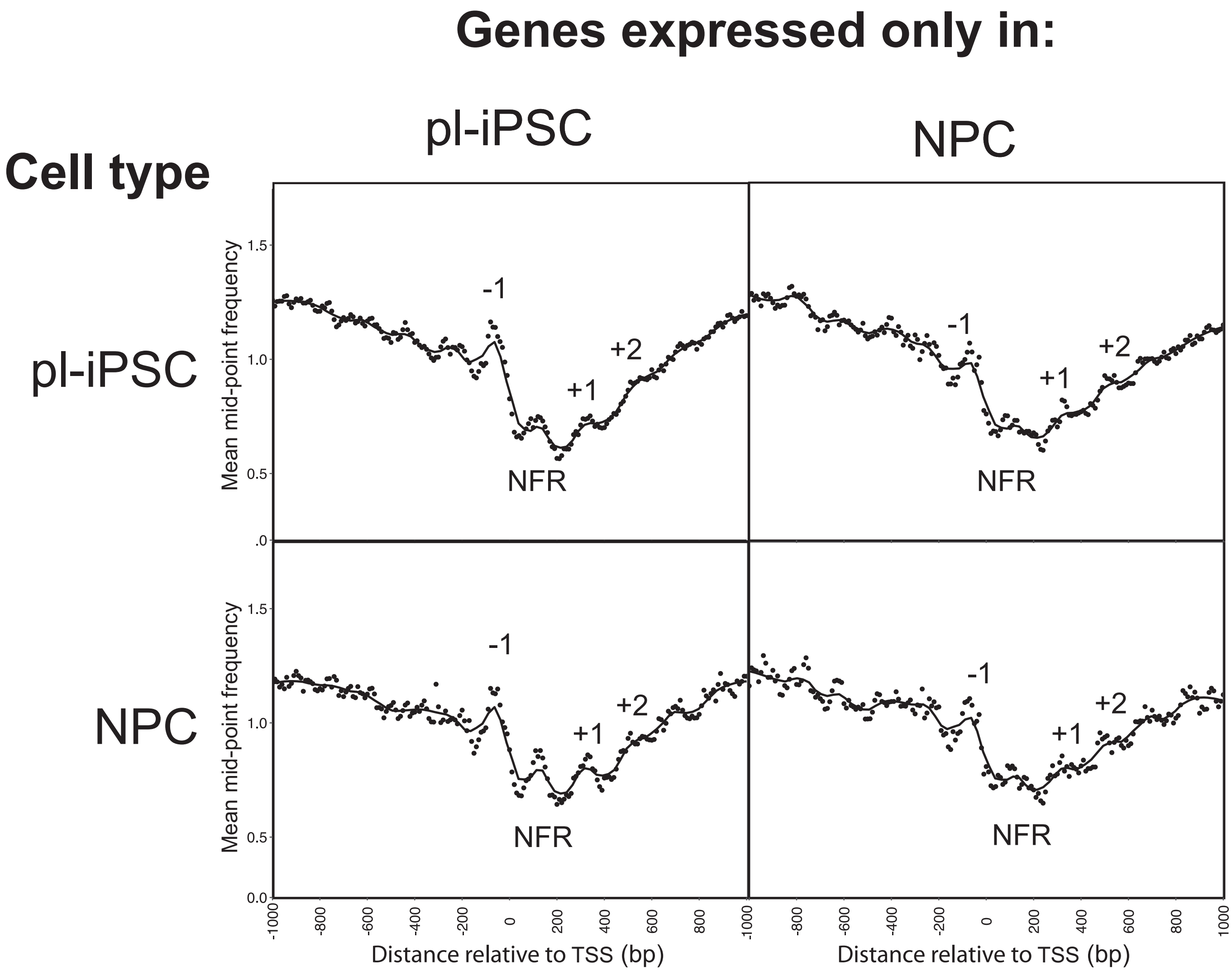

Figure EV3. Nucleosome distributions at the TSS of genes uniquely expressed in either pluripotent or NPC in both active and inactive cell states.
Global frequency distribution of nucleosome distributions within ± 300 bp of a TSS of genes selected for expression exclusively in pluripotent (pl-iPSC, *n* = 3,833) or NPC (*n* = 2,082), shown for both active and inactive cell states. The features corresponding to the nucleosome-free region (NFR) and −1 to +2 nucleosomes are marked. No correlation between gene activity and positioned nucleosomes was observed.

© 2019 The Authors

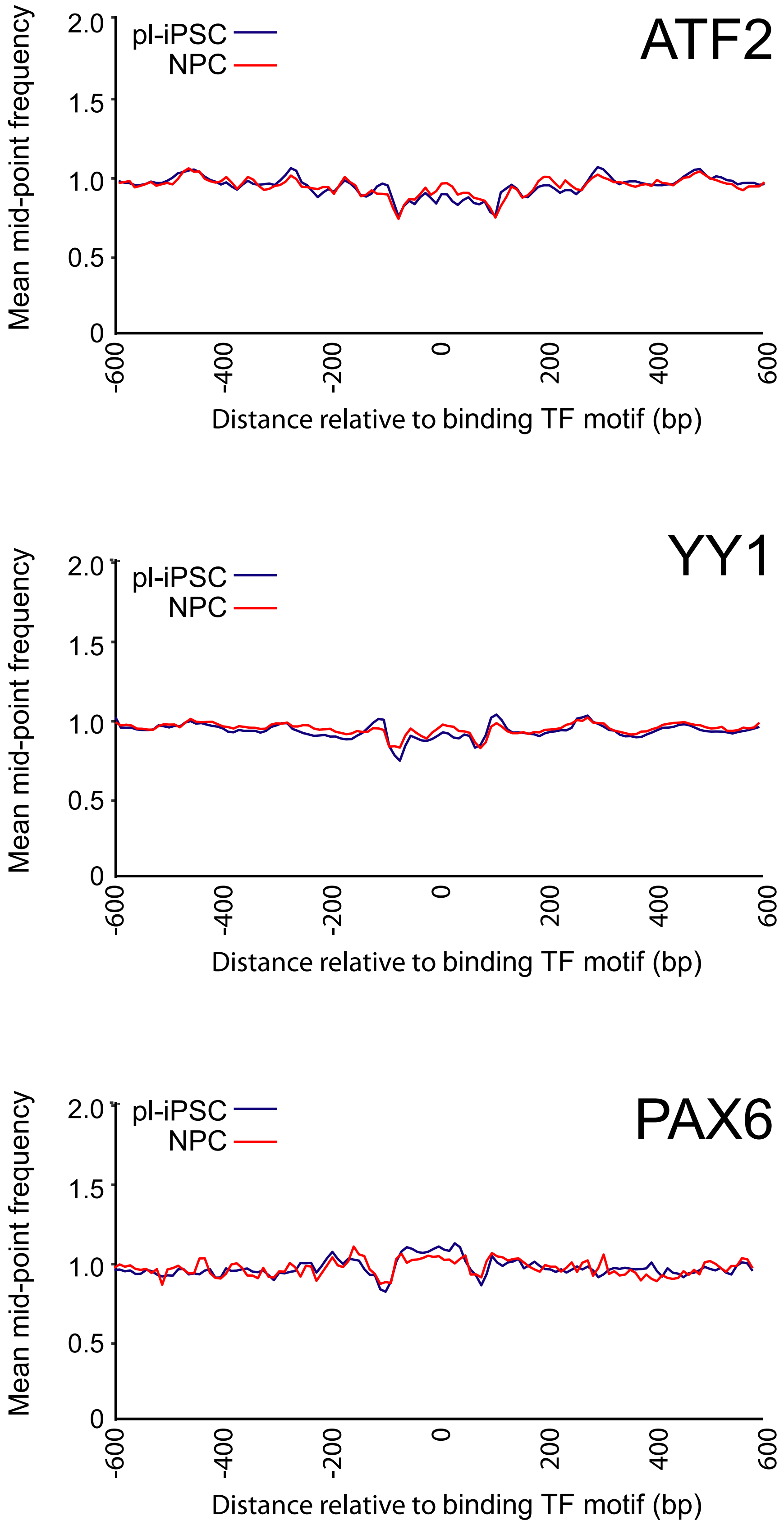

Figure EV4. Nucleosomes are not positioned at several transcription factor binding sites that are involved in neurodevelopment.

Average frequency distributions for sequence mid-point data at and surrounding transcription factor binding sites (± 600 bp) for nucleosomes at ATF2 (*n* = 9,881), YY1 (*n* = 39,945) and PAX6 (*n* = 1,432) sites.

© 2019 The Authors

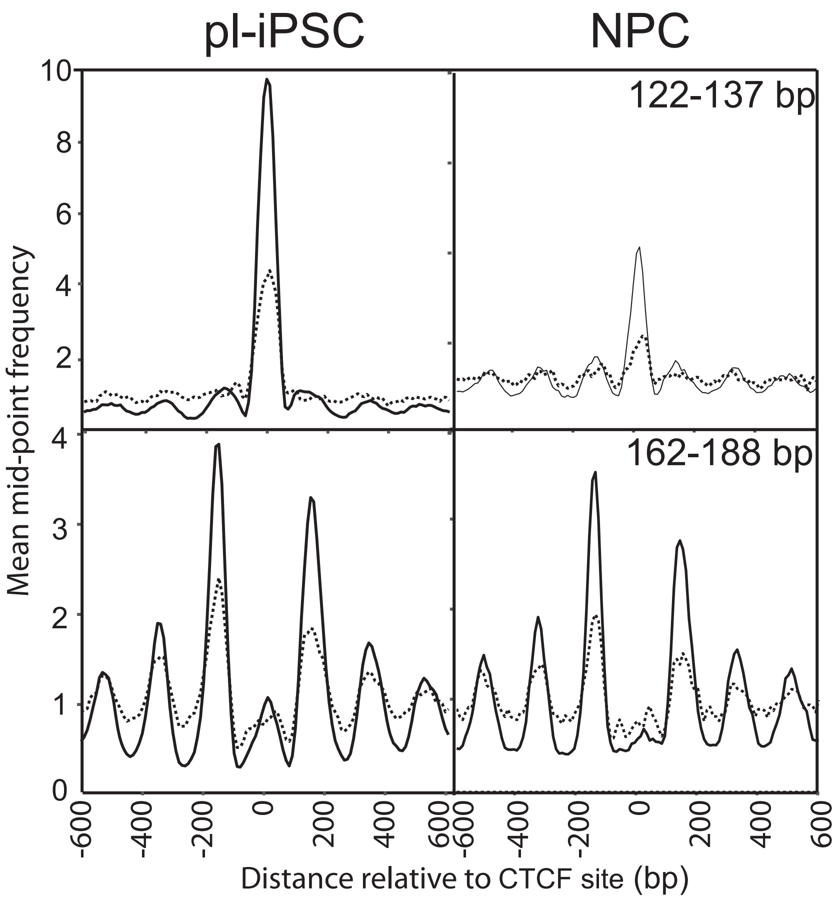

Figure EV5. Cluster analysis of CTCF complexes and nucleosome positions.
Cluster analysis of nucleosome positioning centred on the CTCF site ± 300 bp. CTCF sites were clustered based on the 122–137 bp mid-point sequence read values (CTCF protein complex) from pl-iPSC, shown as a frequency distribution in upper left panel. The frequency distribution data for the same sites were plotted using 122–137 bp data from NPC (upper right panel) and 162–188 bp data (nucleosomes).

© 2019 The Authors
