# Supplementary material for: Nucleosome dynamics of human iPSC during neural differentiation
Source: EMBO Rep. 2019 Apr 29;20(6):e46960. doi: 10.15252/embr.201846960 (PMC6549019; doi:10.15252/embr.201846960)
Supplement: Supplementary file 2 — Table EV1 [file EMBR-20-e46960-s002.docx]

**Table EV1: Numbers of highly positioned nucleosomes detected.**

| **Cell type** | **No. of nucleosomes** |
| --- | --- |
| pl-iPSC | 48,840 |
| NPC | 408,152 |
| K562 | 363,784 |
| GM12878 | 241,064 |
