# Supplementary material for: Nucleosome dynamics of human iPSC during neural differentiation
Source: EMBO Rep. 2019 Apr 29;20(6):e46960. doi: 10.15252/embr.201846960 (PMC6549019; doi:10.15252/embr.201846960)
Supplement: Supplementary file 3 — Table EV2 [file EMBR-20-e46960-s003.docx]

**Table EV2**: **Distribution of positioned nucleosomes from pluripotent iPSC (pl-iPSC) and NPC across 15 chromatin states**

| **Chromatin** | **No. of nucleosomes** | | **% of total** | |
| --- | --- | --- | --- | --- |
| **State** | **pl-iPSC** | **NPC** | **pl-iPSC** | **NPC** |
| 1 | 842 | 1,691 | 1.73 | 0.41 |
| 2 | 106 | 153 | 0.22 | 0.04 |
| 3 | 15 | 9 | 0.03 | 0 |
| 4 | 677 | 1,672 | 1.39 | 0.41 |
| 5 | 5,917 | 58,742 | 12.14 | 14.41 |
| 6 | 37 | 82 | 0.08 | 0.02 |
| 7 | 2,108 | 5,241 | 4.32 | 1.29 |
| 8 | 2,096 | 3,732 | 4.3 | 0.92 |
| 9 | 2,729 | 7,010 | 5.6 | 1.72 |
| 10 | 59 | 21 | 0.12 | 0.01 |
| 11 | 83 | 69 | 0.17 | 0.02 |
| 12 | 95 | 47 | 0.19 | 0.01 |
| 13 | 264 | 372 | 0.54 | 0.09 |
| 14 | 922 | 4,970 | 1.89 | 1.22 |
| 15 | 32,802 | 323,955 | 67.28 | 79.45 |
| **Total** | **48,752** | **407,766** | **100** | **100** |

Distribution of positioned nucleosomes across chromatin states of pluripotent and NPC cell types developed from H1 ES cells [1]. Chromatin states are based on the 15-state model of Ernst and Kellis [2]. The Table shows the calculated numbers and their percentage of the total number of positioned nucleosomes.

# Roadmap Epigenomics Consortium (2015) Integrative analysis of 111 reference human epigenomes. Nature, 518: 317–330

1. Ernst J, Kellis M. ChromHMM: automating chromatin-state discovery and characterization. Nature Methods, 9:215-216, 2012.
