# Supplementary material for: Nucleosome dynamics of human iPSC during neural differentiation
Source: EMBO Rep. 2019 Apr 29;20(6):e46960. doi: 10.15252/embr.201846960 (PMC6549019; doi:10.15252/embr.201846960)
Supplement: Supplementary file 4 — Table EV3 [file EMBR-20-e46960-s004.docx]

**Table EV3: Chromatin state calculations for nucleosomes**

|  | Pl-iPS | default | p(default) | expected | O-E | O-E/E | O-E/E*100 |
| --- | --- | --- | --- | --- | --- | --- | --- |
| 1_Active_Promoter | 315 | 9649596 | 0.00 | 173.92 | 141.08 | 0.81 | 81.11 |
| 2_Weak_Promoter | 547 | 17972821 | 0.01 | 323.94 | 223.06 | 0.69 | 68.86 |
| 3_Poised_Promoter | 188 | 18382770 | 0.01 | 331.33 | -143.33 | -0.43 | -43.26 |
| 4_Strong_Enhancer | 129 | 2625615 | 0.00 | 47.32 | 81.68 | 1.73 | 172.59 |
| 5_Strong_Enhancer | 380 | 7027601 | 0.00 | 126.67 | 253.33 | 2.00 | 200.00 |
| 6_Weak_Enhancer | 1517 | 34587847 | 0.01 | 623.41 | 893.59 | 1.43 | 143.34 |
| 7_Weak_Enhancer | 1312 | 65853579 | 0.02 | 1186.95 | 125.05 | 0.11 | 10.54 |
| 8_Insulator | 2725 | 21846871 | 0.01 | 393.77 | 2331.23 | 5.92 | 592.03 |
| 9_Txn_Transition | 551 | 25543545 | 0.01 | 460.40 | 90.60 | 0.20 | 19.68 |
| 10_Txn_Elongation | 591 | 81202908 | 0.03 | 1463.61 | -872.61 | -0.60 | -59.62 |
| 11_Weak_Txn | 7385 | 496238486 | 0.19 | 8944.23 | -1559.23 | -0.17 | -17.43 |
| 12_Repressed | 530 | 37571070 | 0.01 | 677.18 | -147.18 | -0.22 | -21.73 |
| 14_Repetitive/CNV | 1201 | 3946701 | 0.00 | 71.14 | 1129.86 | 15.88 | 1588.33 |
| 15_Repetitive/CNV | 2817 | 2216760 | 0.00 | 39.95 | 2777.05 | 69.50 | 6950.43 |
| 13_Heterochrom/lo | 28107 | 1854808938 | 0.69 | 33431.17 | -5324.17 | -0.16 | -15.93 |
