# Supplementary material for: Nucleosome dynamics of human iPSC during neural differentiation
Source: EMBO Rep. 2019 Apr 29;20(6):e46960. doi: 10.15252/embr.201846960 (PMC6549019; doi:10.15252/embr.201846960)
Supplement: Supplementary file 5 — Table EV4 [file EMBR-20-e46960-s005.docx]

**Table EV4: Chromatin state calculations for CTCF sites.**

| Chromatin state | Observed | p(default) | Expected |
| --- | --- | --- | --- |
| 1_Active_Promoter | 160 | 0.003601301 | 34 |
| 2_Weak_Promoter | 446 | 0.00670759 | 64 |
| 3_Poised_Promoter | 437 | 0.006860586 | 65 |
| 4_Strong_Enhancer | 25 | 0.000979899 | 9 |
| 5_Strong_Enhancer | 66 | 0.002622753 | 25 |
| 6_Weak_Enhancer | 748 | 0.012908441 | 123 |
| 7_Weak_Enhancer | 167 | 0.024577045 | 234 |
| 8_Insulator | 6331 | 0.008153414 | 78 |
| 9_Txn_Transition | 337 | 0.009533041 | 91 |
| 10_Txn_Elongation | 134 | 0.030305528 | 288 |
| 11_Weak_Txn | 149 | 0.185199887 | 1762 |
| 12_Repressed | 39 | 0.014021802 | 133 |
| 13_Heterochrom/lo | 415 | 0.001472938 | 14 |
| 14_Repetitive/CNV | 21 | 0.000827311 | 8 |
| 15_Repetitive/CNV | 3 | 0.692228464 | 6587 |
|  |  |  |  |
| TOTAL | 9478 | 1 | 9516 |
|  |  |  |  |
| number of CTCF sites = 9516 |  |  |  |
