# Supplementary material for: Nucleosome dynamics of human iPSC during neural differentiation
Source: EMBO Rep. 2019 Apr 29;20(6):e46960. doi: 10.15252/embr.201846960 (PMC6549019; doi:10.15252/embr.201846960)
Supplement: Supplementary file 6 — Table EV5 [file EMBR-20-e46960-s006.docx]

**Table EV5: Consensus transcription factor binding motif sequences used in this study.**

| Transcription factor | Consensus binding motif | Motif length |
| --- | --- | --- |
| ATF2 | [T][G][A][C][G][T][C][A] | 8 |
| YY1 | [A][A][G\|A\|C][A][T][G][G][C][G\|C\|T][G\|C\|A][C] | 11 |
| PAX6 | [A][T][T][C][A][T][G][C][A\|C\|G\|T][T][G][A] | 12 |
| CTCF | [C][C][A\|G][C\|G][C\|T][A][G][G\|A][T\|G][G][G][C\|T] | 12 |
| RE1 | [A\|C\|G\|T][T][T\|C][A][G][A\|C][A\|G][C][C][A\|C\|G\|T]  [A\|C\|G\|T][A\|G][G][A\|C][G\|C][A][G] | 17 |
